# Metastatic colon cancer derived from a diverticulum incidentally found at herniorrhaphy: a case report

**DOI:** 10.1186/s40792-018-0455-y

**Published:** 2018-05-15

**Authors:** Jiro Kimura, Alan Kawarai Lefor, Shota Fukai, Kentaro Yoshikawa, Shingo Sasamatsu, Takashi Sakamoto, Ken Mizokami, Masaki Kanzaki, Tadao Kubota, Akira Saito, Hiroshi Izumi, Kunpei Honjo, Kunihiko Nagakari, Masaki Fukunaga

**Affiliations:** 1 Department of Surgery, Tokyo Bay Urayasu Ichikawa Medical Center, 3-4-32, Todaijima, Urayasu, Chiba 279-0001 Japan; 2 Department of Surgery, Jichi Medical University, 3311-1 Yakushiji, Shimotsuke-City, Tochigi 329-0498 Japan; 3 Department of Pathology, Tokyo Bay Urayasu Ichikawa Medical Center, Chiba, Japan; 4 Department of Surgery, Juntendo Urayasu Hospital, Juntendo University, Chiba, Japan

**Keywords:** Colon cancer, Diverticulum, Hernia sac

## Abstract

**Background:**

There are few reports of metastases from colon cancer to an inguinal hernia sac, and few reports of colon cancer originating in diverticula. We report a patient with carcinoma of the sigmoid colon arising in two diverticula, who presented with peritoneal seeding to an inguinal hernia sac, and a review of the literature.

**Case presentation:**

A 55-year-old male underwent open herniorrhaphy for a left inguinal hernia. At operation, a nodule in the inguinal hernia sac was resected and histologic examination revealed adenocarcinoma, which was suspected to be a metastasis from a distant primary lesion. Postoperative evaluation included colonoscopy and positron emission tomography which showed two suspected lesions in sigmoid diverticula. Laparoscopic subtotal colectomy was performed, and pathology revealed adenocarcinoma in two sigmoid diverticula.

**Conclusions:**

If a nodule is found in an inguinal hernia sac, especially in older patients, peritoneal metastases should be considered. Resection of the nodule with histopathologic evaluation is essential. Colon cancer arising in a diverticulum should be considered as a possible site of the primary lesion.

## Background

Peritoneal seeding from a cancer of unknown origin may be encountered and is usually found incidentally during open or laparoscopic abdominal surgery. After evaluation to identify the primary tumor, some are found and the others remain unknown.

Malignant tumors presenting in an inguinal hernia sac are extremely rare. In 1959, Yoell reported that routine histological examination of resected hernia sacs revealed approximately 0.4% contained malignant lesions [1]. There are few reports of metastases from colon cancer to an inguinal hernia sac. There are also few reports of colon cancer arising within diverticula. To the best of our knowledge, this is the first report of a patient with a peritoneal metastasis in an inguinal hernia sac from sigmoid colon cancer arising in diverticula.

## Case presentation

**Fig. 1:**
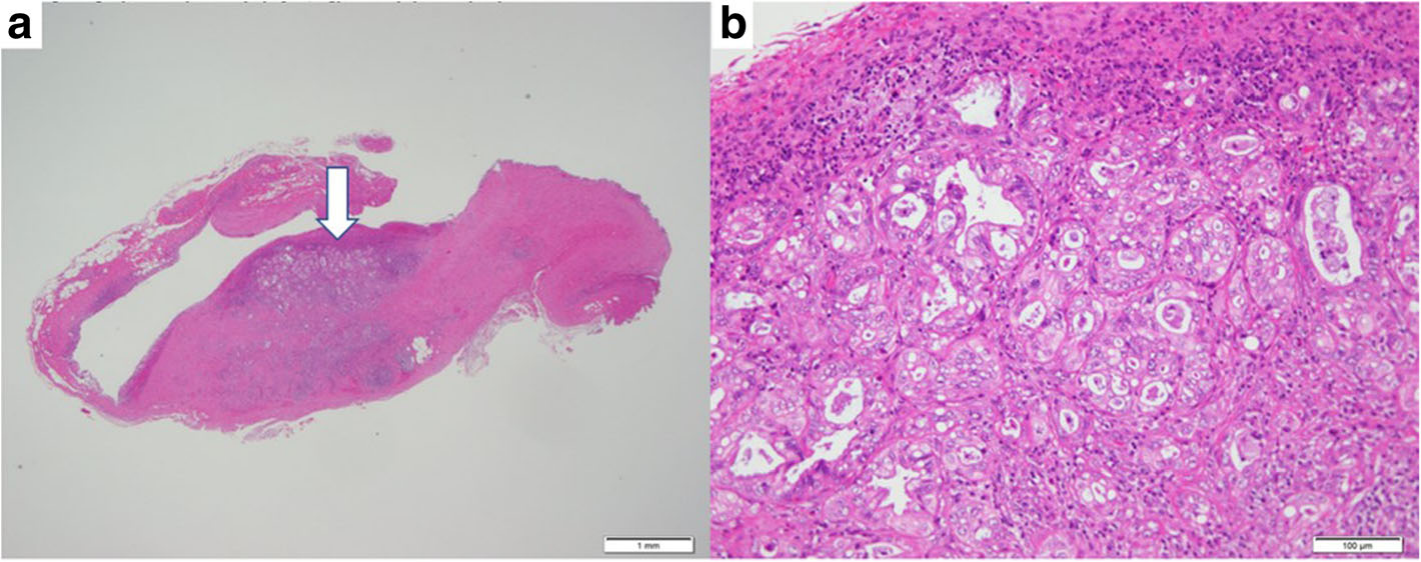
**a** The nodule inside the hernia sac revealed moderately differentiated adenocarcinoma (arrow). **b** high power view of the same lesion shown in (**a**)

**Fig. 2:**
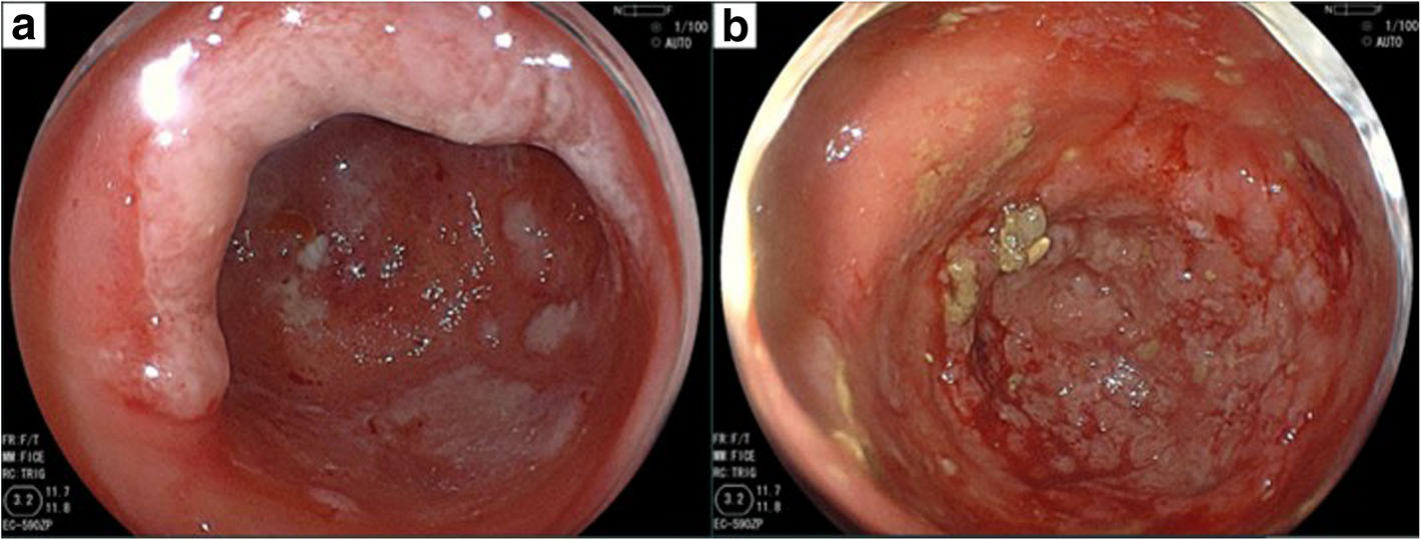
Colonoscopy showed two diverticula of the sigmoid colon containing elevated lesions. **a** The proximal lesion had erythema with marginal erosion. **b** The distal lesion had irregularly elevated mucosa

**Fig. 3:**
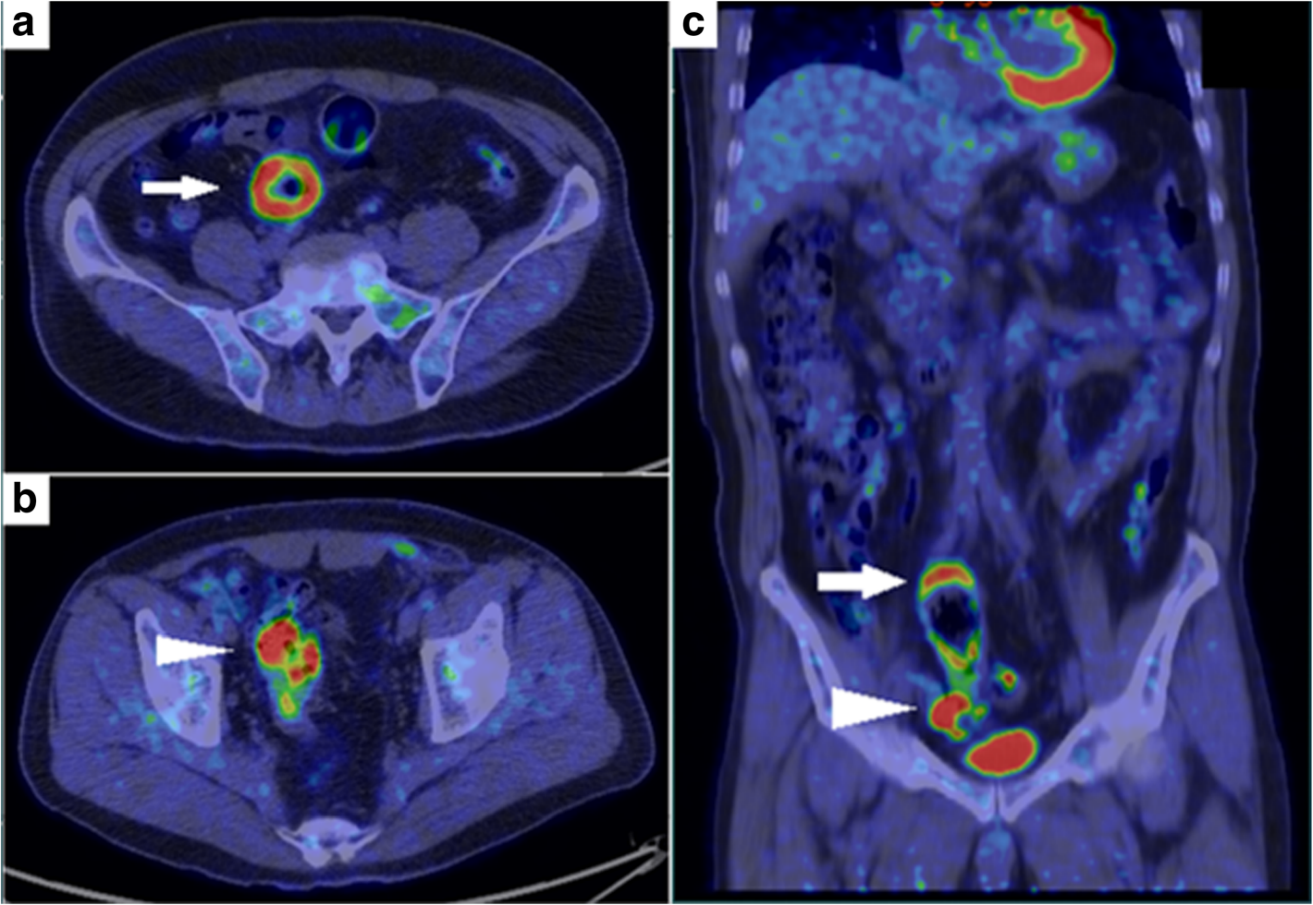
Positron emission tomography-computed tomography revealed two lesions in the sigmoid colon with increased maximal standardized uptake values (SUV_max_) (arrow). **a** axial view: the proximal circumferential lesion (arrow) with SUV_max_ = 10.7, **b** axial view: the distal lesion (arrowhead) with SUV_max_ = 11.2, and **c** coronal view: both lesions located in the sigmoid colon

**Fig. 4:**
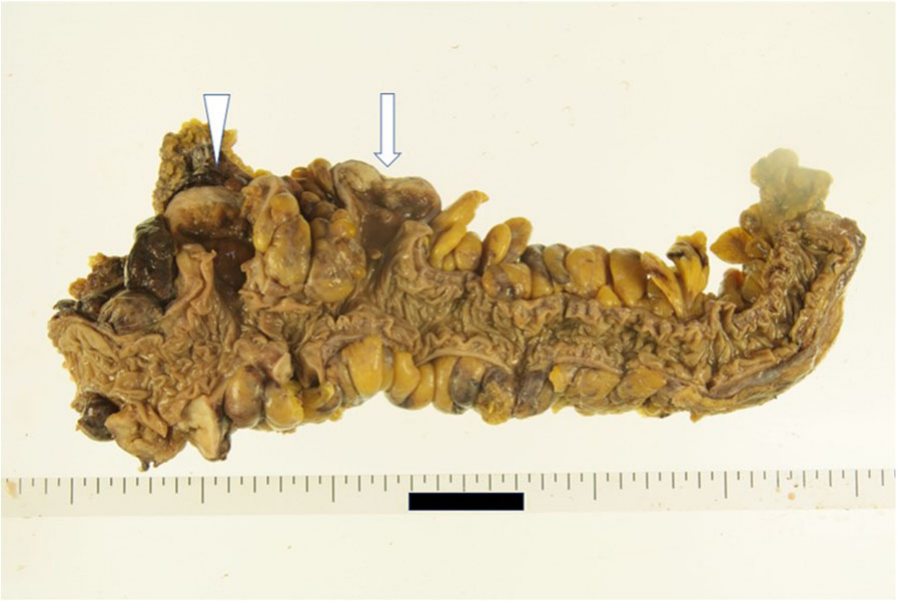
The specimen of the sigmoid colon revealed the proximal lesion (arrow) and the distal lesion (arrowhead). They were within the diverticula

**Fig. 5:**
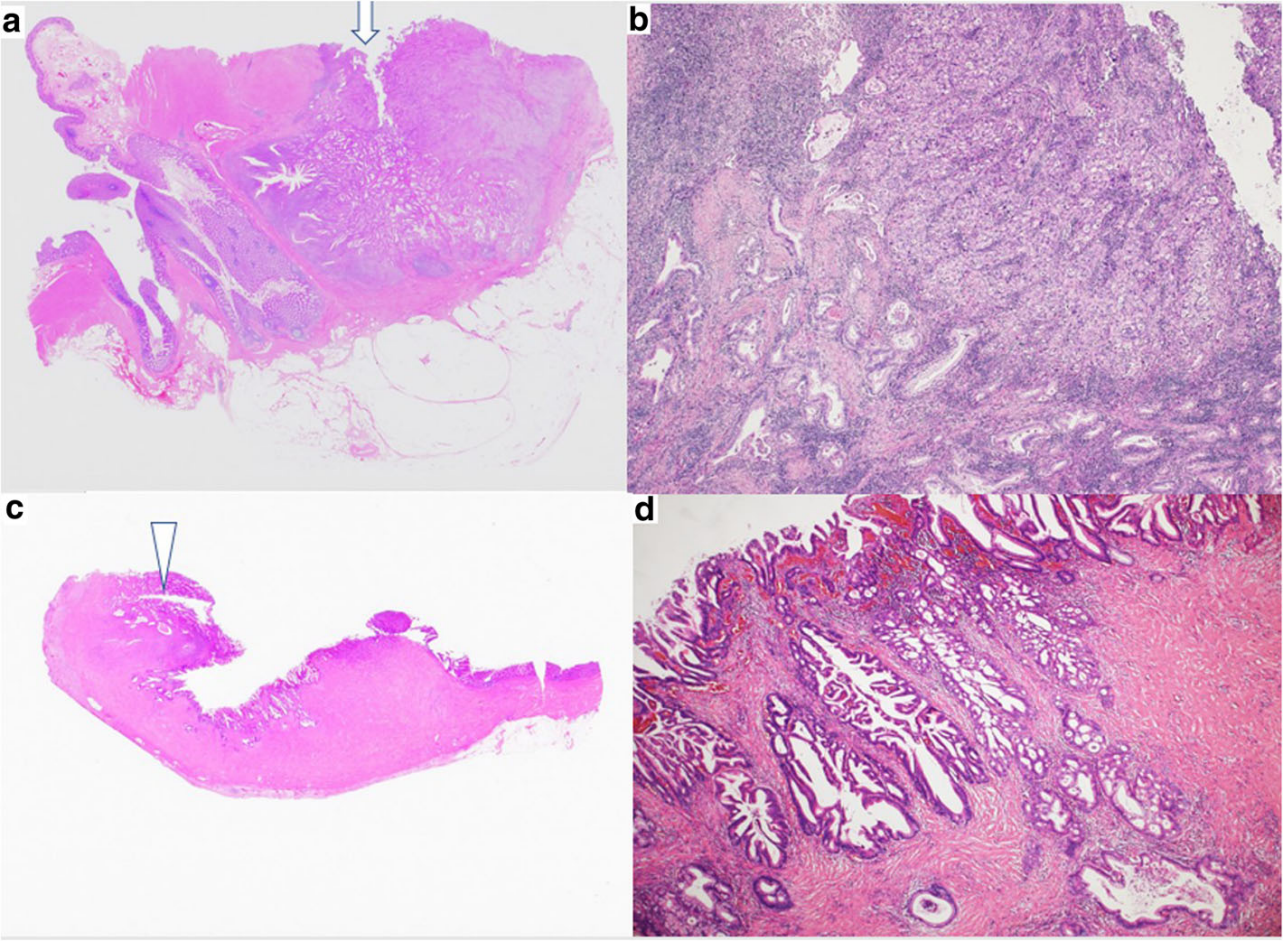
**a** The pathology of the proximal lesion within diverticula revealed moderately differentiated adenocarcinomas (arrow). **b** Low power view of the same lesion shown in (**a**). **c** The distal lesion revealed moderately differentiated adenocarcinoma (arrowhead). **d** Low power view of the same lesion shown in (**c**)

A 55-year-old male presented with a 2-week history of a bulge in the left inguinal area which was not painful but caused discomfort. The patient had no significant past medical history. Physical examinations showed that vital signs were normal, and a bulge in the left groin was soft and easily reducible, consistent with an inguinal hernia. Elective hernia repair was performed with the Lichtenstein method 1 week later. During operation, we found a white nodule inside the hernia sac, which measured 10 mm in diameter. The nodule was excised and examined histopathologically. The postoperative course was unremarkable, and he was discharged without complications. Examination of the nodule revealed moderately differentiated adenocarcinoma (Fig. 1a, b). Immunohistochemistry showed positive CK7, CK20, and AE1/AE3, and negative prostate-specific antigen and calretinin. The nodule clearly represented peritoneal seeding from an adenocarcinoma of unknown primary. Ultrasound, computed tomography (CT) scan, and esophagogastroduodenoscopy showed no obvious site of the primary tumor. Colonoscopy did not show any typical findings of colon cancer, but there were many diverticula in the sigmoid colon, two of which contained mucosal erosions or inflammatory lesions. These lesions each measured 2 cm in diameter (Fig. 2a, b,). Biopsies of the lesions were negative, and repeat biopsy was also negative for malignancy. A positron emission tomography-computed tomography (PET-CT) scan revealed two lesions in the sigmoid colon, with maximum standardized uptake values elevated to 10.7 and 11.2, respectively (Fig. 3a–c). Sigmoid colon cancer was considered the most likely diagnosis. Laparoscopic sigmoid colon resection was performed. At operation, no other metastatic lesions were found. In the specimen, the two lesions seen at endoscopy were identified (Fig. 4). The final pathology of both lesions revealed moderately differentiated adenocarcinomas, within diverticula in the sigmoid colon (Fig. 5a–d) which histologically appeared the same as the nodule found in the hernia sac.

## Discussion

Despite advances in the treatment of patients with a variety of malignancies, colon cancer with peritoneal metastases is still associated with a poor prognosis. Watanabe et al. reported that 5% of patients with colon cancer have peritoneal metastases at the time of diagnosis [2]. They also found that synchronous peritoneal metastases are associated with a poor prognosis. The present patient had peritoneal metastases at the time of an inguinal hernia repair, and the site of the primary tumor was unknown until after the colon resection. Cancer of unknown origin represents 3 to 5% of all malignant epithelial tumors [3]. Adenocarcinoma comprises approximately 70% of cancers of unknown origin. In an autopsy series, tumors of the lung, pancreas, hepatobiliary tree, and kidney account for approximately two thirds of cases of cancer of unknown primary [4]. Colon cancer is relatively rare as a site of these tumors. Colonoscopy and biopsy did not reveal the definitive diagnosis in this patient. However, PET-CT scan identified the colon as the likely origin of the peritoneal metastases, which was confirmed after resection of the colon.

Only four patients with metastatic colon cancer in an inguinal hernia sac have been reported. All of these patients were males over 60 years of age. It remains uncertain why metastases develop within a hernia sac. Roslyn et al. suggested that the mechanism might be explained by local inflammation of the sac and gravity, in a manner similar to drop metastases to the pelvic cul-de-sac [5]. Yu et al. demonstrated that inflammatory cytokines (interleukin-1β and tumor necrosis factor-α) enhanced tumor cell adhesion in biological tests [6]. The present patient did not have any trauma or previous left inguinal surgery to suggest a cause of inflammation. We hypothesize that the sigmoid colon, including the tumors in the diverticula, may have descended in the hernia sac and may have contributed to inflammation and cancer seeding. It is important to make an effort to identify the primary lesion when a metastatic nodule is found during inguinal hernia repair. However, routine studies do not have to be done before the metastasis is examined histologically because this is very rare.

Malignancies can arise in colonic diverticula. The diagnosis was made based on the endoscopic finding of a tumor within a diverticulum [7]. However, establishing the diagnosis may be complicated by abnormal findings, such as abscess formation, submucosal progression, or diverticulitis [8]. Hence, endoscopic findings may not facilitate establishing the definitive diagnosis, as in the present patient.

There are 11 previous reports of colon cancer arising in a diverticulum. Of these, nine were in an advanced at the time of diagnosis. Eight of these lesions were in the left colon. Since colonic diverticula are thin-walled and lack a muscular layer, cancers arising within a diverticulum can easily penetrate the serosa which may facilitate early development of disseminated disease. Patients with cancers arising in a diverticulum need careful evaluation and follow-up [8]. The present patient had two separate cancers in sigmoid diverticula which were considered synchronous, because the pathological characteristics were different. The proximal lesion was mainly moderately differentiated adenocarcinoma, accompanied by highly and poorly differentiated adenocarcinoma. However, the distal lesion was mainly moderately differentiated adenocarcinoma without poorly differentiated adenocarcinoma. To the best of our knowledge, the present case is the first report of two cancers in colonic diverticula.

## Conclusions

If a nodule is found in an inguinal hernia sac, especially in older patients, peritoneal metastasis should be considered. Resection of the nodule with histopathologic evaluation is essential. Colon cancer arising in a diverticulum should be considered as a possible site of the primary lesion.

## Abbreviations

CT: Computed tomography
PET: Positron emission tomography
SUV_max_: Maximum standardized uptake value
